# MAXWELL A. POLLACK AWARD LECTURE

**DOI:** 10.1093/geroni/igz038.722

**Published:** 2019-11-08

**Authors:** Robert Harootyan

**Affiliations:** Senior Service America Inc., Silver Spring, Maryland, United States

## Abstract

The Maxwell A. Pollack Award for Productive Aging recognizes instances of practice informed by research and analysis, research that directly improved policy or practice, and distinction in bridging the worlds of research and practice. The award lecture will be presented by the 2018 recipient, Karen Fredriksen-Goldsen, PhD, University of Washington. This award is generously funded by The New York Community Trust.

